# P-378. Benchmarking Healthcare-Associated Infections in Developing Countries Post-COVID-19: From High-Resource Hospitals in Southwest Brazil to Remote Hospitals in Jungle Regions

**DOI:** 10.1093/ofid/ofae631.579

**Published:** 2025-01-29

**Authors:** Bráulio R G M Couto, Carlos E Starling, Hoberdan Pereira, Estevão Urbano Silva, Ana Paula Ladeira, Rossana Souza, Aline Bentes, Bruna De Souza, Camila Araújo, Cinthia Carolini De Jesus, Claudia M De Oliveira, Daniela Pereira, Diene Pereira, Dilma Silva, Eder Munefiça, Emilly Vieira, Ewaldo Mattos Junior, Felipe De Paula, Fernando Carvalho, Graziela Pereira, Guenael Freire Souza, Guilherme da Cruz, Hellen Dourado, herbert Fernandes, Isabela Castro, Karina Versiani, Louranny Cristina Góis, Luana Silveira, Luciana Covello, Luciano Fernandes, Marcelo Esteves, Maria Clara Bueno, Mariana Melo, Raynara Sampaio, Shirley Valadares, Tereza Pereira, Thaís Couto, Virginia Andrade, Walisson Ferreira Carvalho, Naísses Zóia Lima

**Affiliations:** AMECI – Associação Mineira de Epidemiologia e Controle de Infecções, Belo Horizonte, Minas Gerais, Brazil; Sociedade Mineira de Infectologia - SMI, Belo Horizonte, Minas Gerais, Brazil; Hospital Municipal Odilon Behrens, Belo Horizonte, Minas Gerais, Brazil; Hospital Madre Teresa, Belo Horizonte, Minas Gerais, Brazil; Biobyte Tecnologia em Epidemiologia, Belo Horizonte, Minas Gerais, Brazil; Biobyte Sistemas, Belo Horizonte, Minas Gerais, Brazil; Hospital Infantil João Paulo II, Belo Horizonte, Minas Gerais, Brazil; Hospital Nossa Senhora das Graças, Sete Lagoas, Minas Gerais, Brazil; Hospital Evangélico, Belo Horizonte, Minas Gerais, Brazil; Hospital Nossa Senhora das Graças, Sete Lagoas, Minas Gerais, Brazil; Santa Casa de Belo Horizonte, Belo Horizonte, Mato Grosso, Brazil; Hospital Nossa Senhora das Graças, Sete Lagoas, Minas Gerais, Brazil; Hospital Evangélico, Belo Horizonte, Minas Gerais, Brazil; Hospital Municipal de Sete Lagoas, Sete Lagoas, Minas Gerais, Brazil; Hospital Regional de Campo Maior, Campo Maior, Piaui, Brazil; Santa Casa Montes Claros, Montes Claros, Minas Gerais, Brazil; Sociedade Mineira de Infectologia, Belo Horizonte, Minas Gerais, Brazil; Biobyte EpiTech, Belo Horizonte, Minas Gerais, Brazil; Hospital Geral de Parauapebas, Parauapebas, Para, Brazil; Hospital Municipal de Sete Lagoas, Sete Lagoas, Minas Gerais, Brazil; Fundação Hospitalar Nossa Senhora de Lourdes, Belo Horizonte, Minas Gerais, Brazil; Santa Casa Montes Claros, Montes Claros, Minas Gerais, Brazil; Santa Casa Montes Claros, Montes Claros, Minas Gerais, Brazil; Hospital Ibiapaba, Barbacena, Minas Gerais, Brazil; Hospital Geral de Parauapebas, Parauapebas, Para, Brazil; Hospital Felício Rocho, Belo Horizonte, Minas Gerais, Brazil; Biocor Instituto – Rede D’Or, Belo Horizonte, Minas Gerais, Brazil; Hospital Nossa Senhora das Graças, Sete Lagoas, Minas Gerais, Brazil; Hospital Evangélico, Belo Horizonte, Minas Gerais, Brazil; Santa Casa Montes Claros, Montes Claros, Minas Gerais, Brazil; Biobyte EpiTech, Belo Horizonte, Minas Gerais, Brazil; Biocor Instituto – Rede D’Or, Belo Horizonte, Minas Gerais, Brazil; Hospital Metropolitana Doutor Célio de Castro, Belo Horizonte, Minas Gerais, Brazil; Hospital Regional de Campo Maior, Campo Maior, Piaui, Brazil; Fundação Hospitalar Nossa Senhora de Lourdes, Belo Horizonte, Minas Gerais, Brazil; Hospital Evangélico, Belo Horizonte, Minas Gerais, Brazil; Hospital Evangélico, Belo Horizonte, Minas Gerais, Brazil; Hospital Madre Teresa, Belo Horizonte, Minas Gerais, Brazil; PUC MInas, Belo Horizonte, Minas Gerais, Brazil; PUC MInas, Belo Horizonte, Minas Gerais, Brazil

## Abstract

**Background:**

Benchmarking is essential for evaluating healthcare quality. In Brazil, few studies on healthcare-associated infections (HAIs) exist, and most focus on internal benchmarking within specific populations, creating a gap in understanding broader regions. Applying benchmarks from high-resource countries to low-resource settings can produce misleading conclusions due to differing healthcare environments. Thus, it's crucial to refine external benchmarks tailored to developing countries.

**Figure 1:**
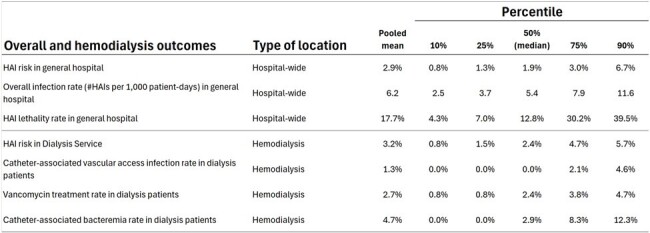
Pooled mean and percentiles (10th, 25th, 50th, 75th, and 90th) for seven indicators related to overall and hemodialysis outcomes.

**Methods:**

The majority of hospitals involved in this study use SACIH+, a hospital infection control software (www.biobytebrasil.com), which compiles data from various Brazilian hospitals. All hospitals followed prospective surveillance protocols for healthcare-associated infections (HAIs) in accordance with the NHSN/CDC guidelines.

Benchmarks for HAIs were established for different categories: Medical/Surgical Intensive Care Unit (ICU) HAI rates, surgical site infections, and hospital-wide HAI rates. These benchmarks were defined as the 10th and 90th percentiles using data collected from 25 hospitals between 2021 and 2023. While not all benchmarks included data from every hospital, each benchmark represents data from at least three hospitals and a minimum denominator of 20.

**Figure 2:**
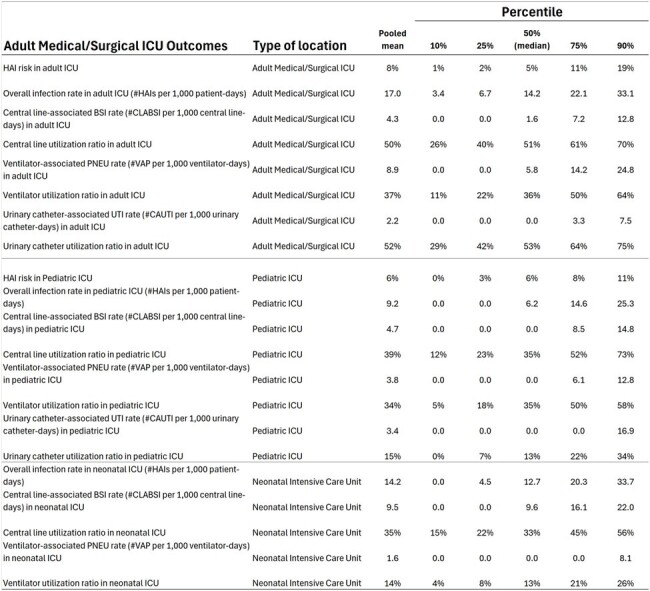
Pooled mean and percentiles (10th, 25th, 50th, 75th, and 90th) for 21 indicators from Medical/Surgical Adult ICU, Pediatric ICU, and Neonatal ICU.

**Results:**

Seven overall and hemodialysis rates were benchmarked (Fig. 1). We included lethality rate and global HAI risk (covering all infection sites) as they are required HAI indicators in Brazil. Figure 2 shows benchmarks for key indicators in Adult Medical/Surgical ICU, Pediatric ICU, and Neonatal ICU. Surgical site infection rates, overall and by surgical service, and for major surgical procedures, are detailed in Figures 3 and 4.

**Figure 3:**
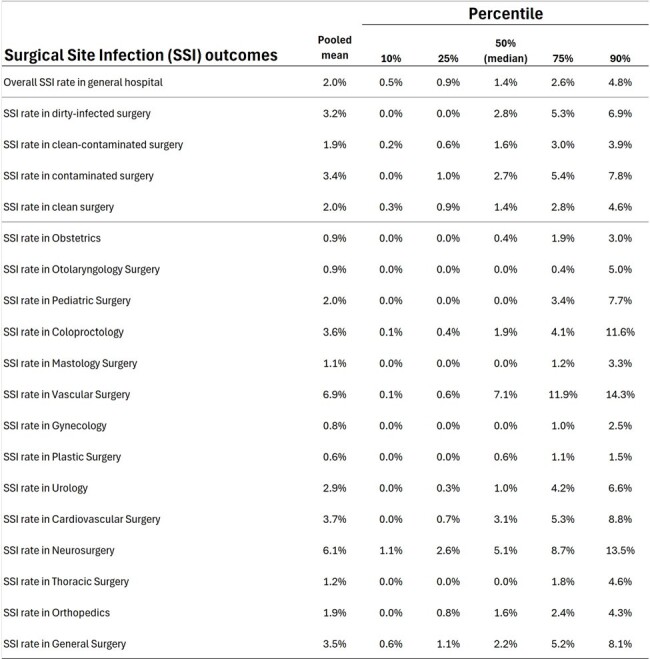
Pooled mean and percentiles (10th, 25th, 50th, 75th, and 90th) for 19 overall surgical site infection rates, stratified by service and wound classification (clean, clean-contaminated, contaminated, and infected).

**Conclusion:**

This study provides a comprehensive summary of benchmarks for 95 key HAI rates applicable to Brazil in the post-COVID-19 era and other developing countries. By offering external benchmarks across diverse settings, including high-resource and remote hospitals, these findings can guide healthcare facilities in improving infection control practices and refining surveillance protocols. Ultimately, the established benchmarks offer a valuable resource for hospitals striving to enhance patient safety and optimize infection control measures globally.

**Figure 4:**
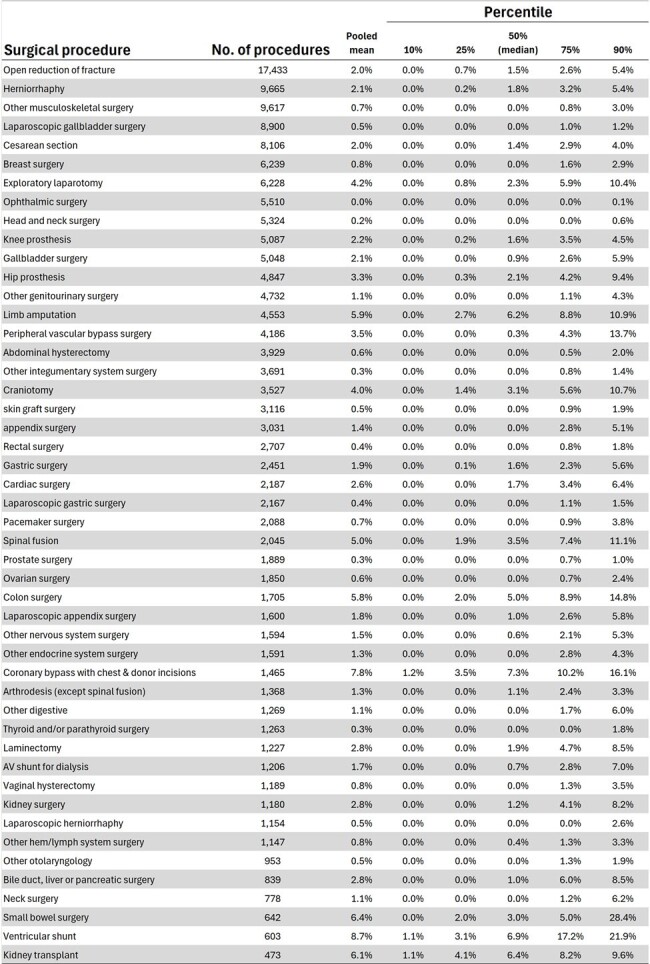
Pooled mean and percentiles (10th, 25th, 50th, 75th, and 90th) for surgical site infections across 48 different surgical procedures.

**Disclosures:**

**All Authors**: No reported disclosures

